# Horizontal (Transverse) Intraarticular Metacarpal Head Fracture in a Young Adult

**DOI:** 10.7759/cureus.16720

**Published:** 2021-07-29

**Authors:** Vasileios K Mousafeiris, Ioannis Papaioannou, Nektaria Kalyva, Georgia Pantazidou, Thomas Repantis

**Affiliations:** 1 Orthopedics, General Hospital of Patras “Agios Andreas”, Patras, GRC; 2 Pediatrics, University Hospital of Patras, Patras, GRC; 3 Otolaryngology - Head and Neck Surgery, General Hospital of Patras “Agios Andreas”, Patras, GRC

**Keywords:** hand injury, metacarpal head fracture, horizontal metacarpal head fracture, transverse metacarpal head fracture, avascular necrosis

## Abstract

Metacarpal head fractures are rare injuries that usually occur during trauma. These fractures are classified into 10 groups, with horizontal (transverse) being the rarest type of fractures. To our knowledge, very few cases have been reported in the literature to date. Here, we present the case of a 21-year-old male who sustained multiple ipsilateral hand injuries accompanied by a horizontal (transverse) fracture of the fourth metacarpal. He underwent open reduction and fixation with Kirschner wires followed by intensive rehabilitation. He finally regained complete active range of motion and grip strength three months after the operation. However, at nine months postoperatively, he developed avascular necrosis, which was asymptomatic and did not need any intervention. Therefore, it is important to maintain a high index of suspicion for possible complications and follow patients regularly, probably even for as long as 12 months after the initial injury.

## Introduction

Metacarpal head fractures are rare and comprise only 4-5% of all metacarpal injuries [1]. Intraarticular fractures of the metacarpal head are even rarer and often require surgical fixation to maintain articular congruence [2-5]. These fractures are classified into 10 groups based on their anatomy, with the horizontal (transverse) fracture pattern being the least common type [3].

Here, we present a rare case of a horizontal (transverse) intraarticular metacarpal head fracture in a 21-year-old male which was sustained during a motorbike accident. In addition to the rarity of the injury and the complexity of the subsequent management, it is crucial to be aware of potential complications that may ensue.

## Case presentation

**Figure 1:**
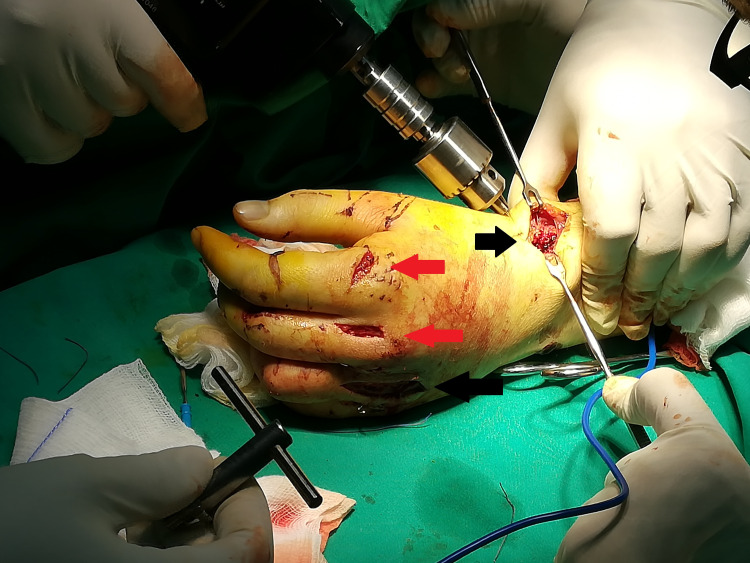
Intraoperative image of the affected hand. The two red arrows show the wounds from the initial injury, while the two black arrows highlight the surgical incisions.

A 21-year-old male patient was admitted to our emergency department due to painful swelling of his left (nondominant) hand after crushing his hand against a wall while riding his motorbike. Both upper extremities were neurovascularly intact. Two small wounds were recognized dorsally over the second and third metacarpophalangeal (MCP) joint (Figure 1).

**Figure 2:**
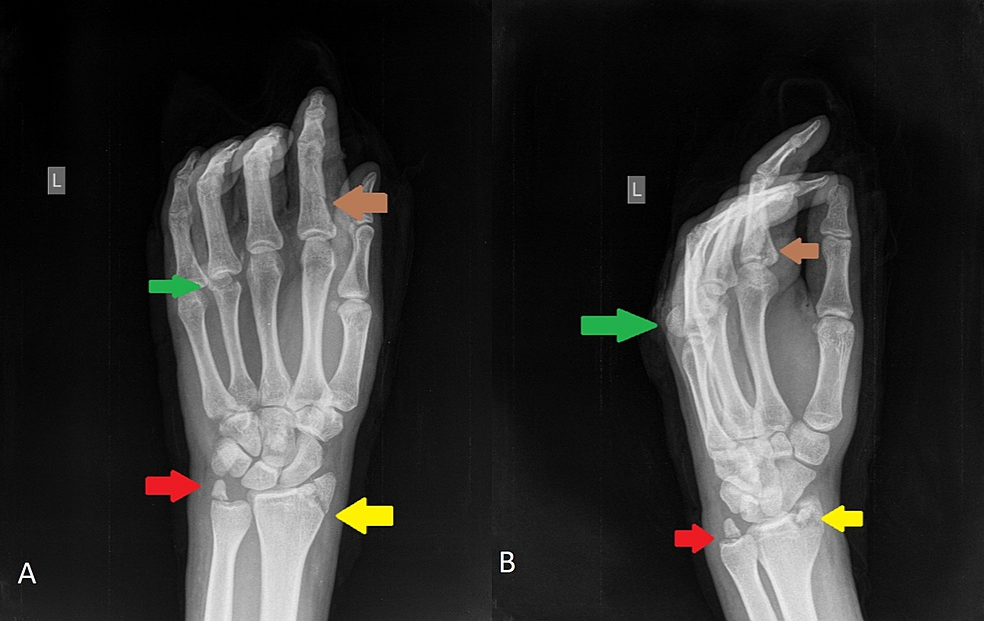
Anteroposterior (A) and profile (B) radiograms of the injured hand and wrist. The brown arrow demonstrates the fracture of the proximal phalanx of the index finger, the red arrow shows the fracture of the styloid process of the ulna, the yellow arrow indicates the fracture of the styloid process of the radius, and the green arrow highlights the fracture of the head of the fourth metacarpal with complete horizontal displacement.

Plain radiographs of the injured hand revealed a nondisplaced fracture of the proximal phalanx of the index finger, a fracture of the styloid process of the ulna, a fracture of the styloid process of the radius, and an uncommon fracture of the head of the fourth metacarpal with complete horizontal displacement of the distal fragment (Figures 2A, 2B).

**Figure 3:**
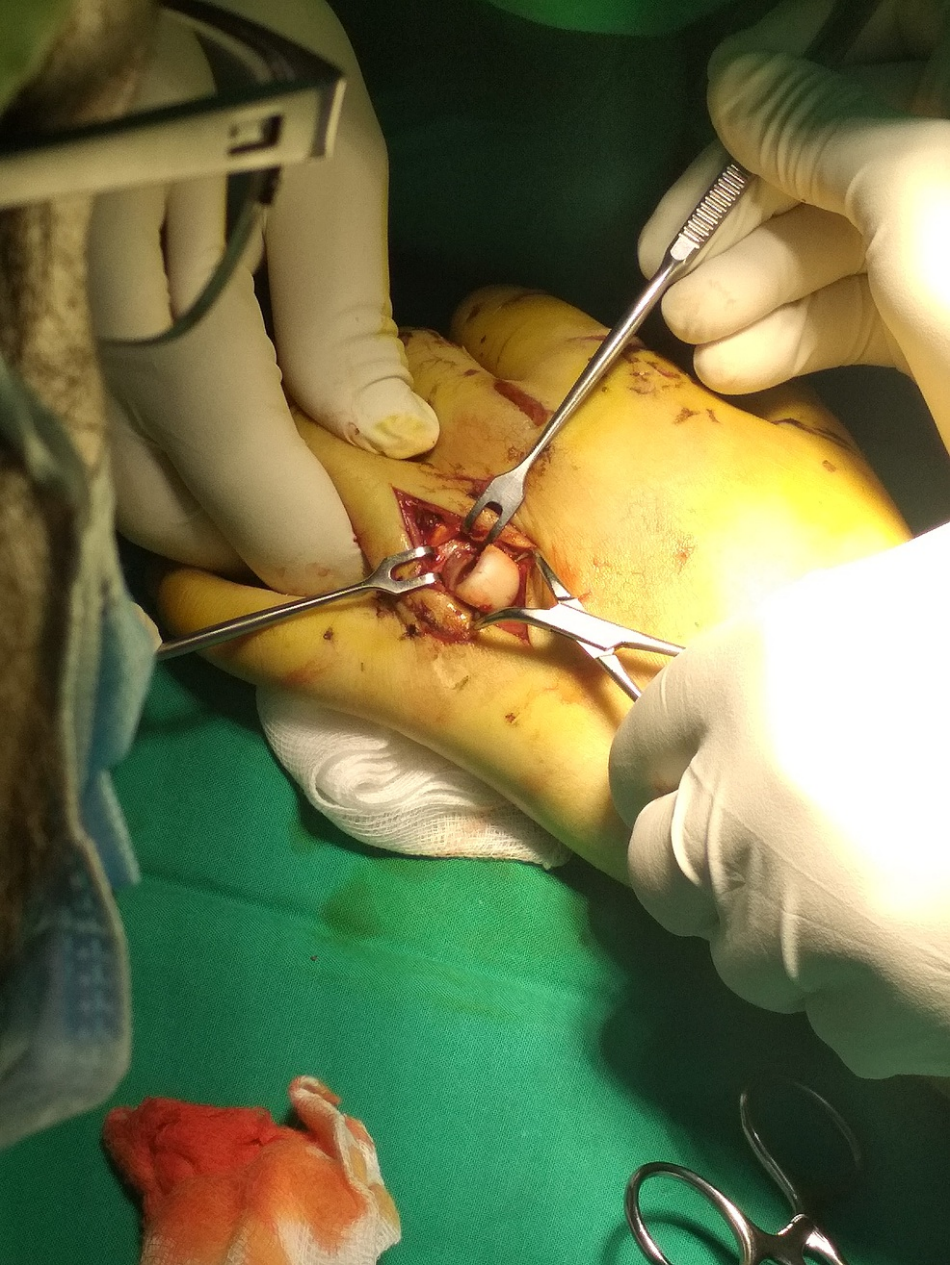
Intraoperative photo demonstrates the horizontal position of the metacarpal head after skin incision and subcutaneous tissue dissection.

The patient consented to surgery, which was performed on the same day. Following a longitudinal dorsal incision and subcutaneous dissection, the metacarpal head was found lying through the extensor tendon. The capsule and the sheath of the extensor tendon were disrupted from the metacarpal head. The tendon was mobilized radially which surprisingly revealed no capsular attachment neither on the volar nor on the dorsal side, whereas the collateral ligaments of the MP joint were intact (Figure 3).

**Figure 4:**
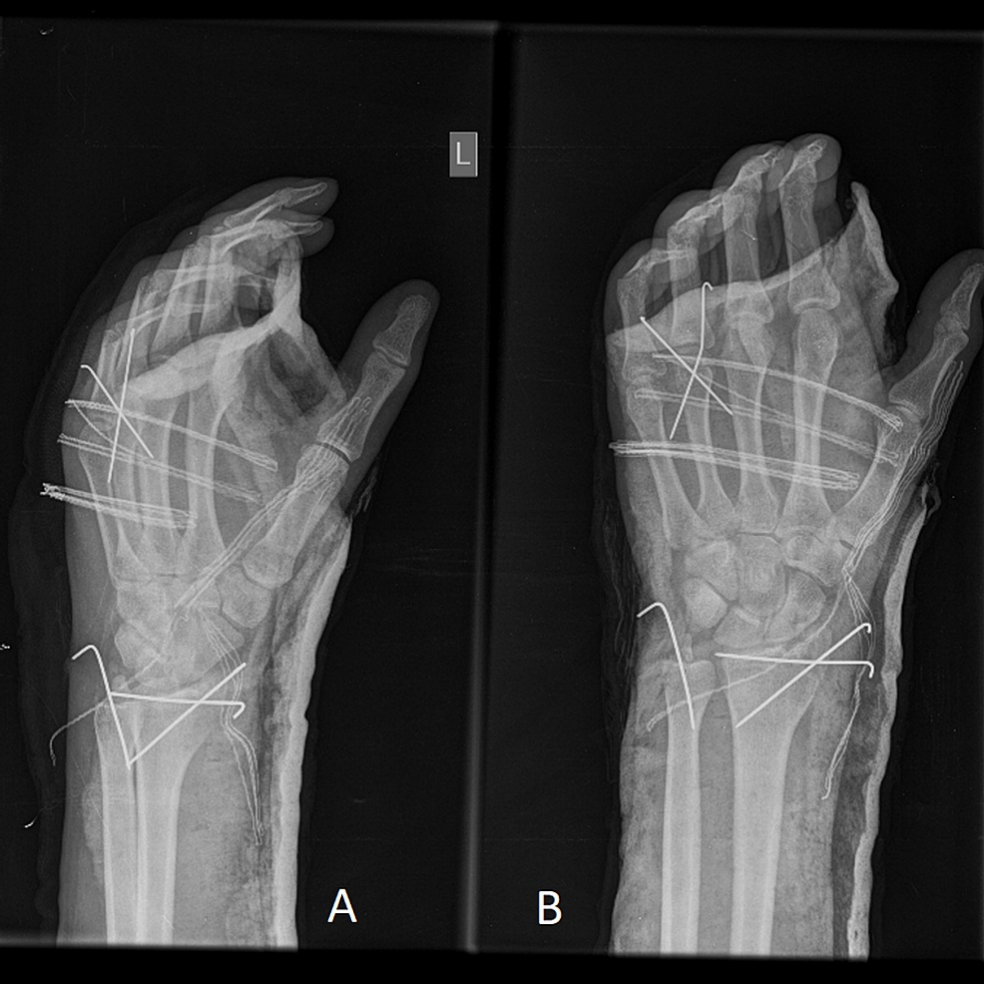
Postoperative profile (A) and anteroposterior (B) radiograms of the hand and wrist demonstrate the final fixation.

The fracture was reduced and stabilized via two Kirschner wires and the capsule and the extensor mechanism were restored. In addition, a wire was placed to the ulna’s styloid process and two wires to the radial styloid process after open reduction of the wrist joint (Figures 4A, 4B).

**Figure 5:**
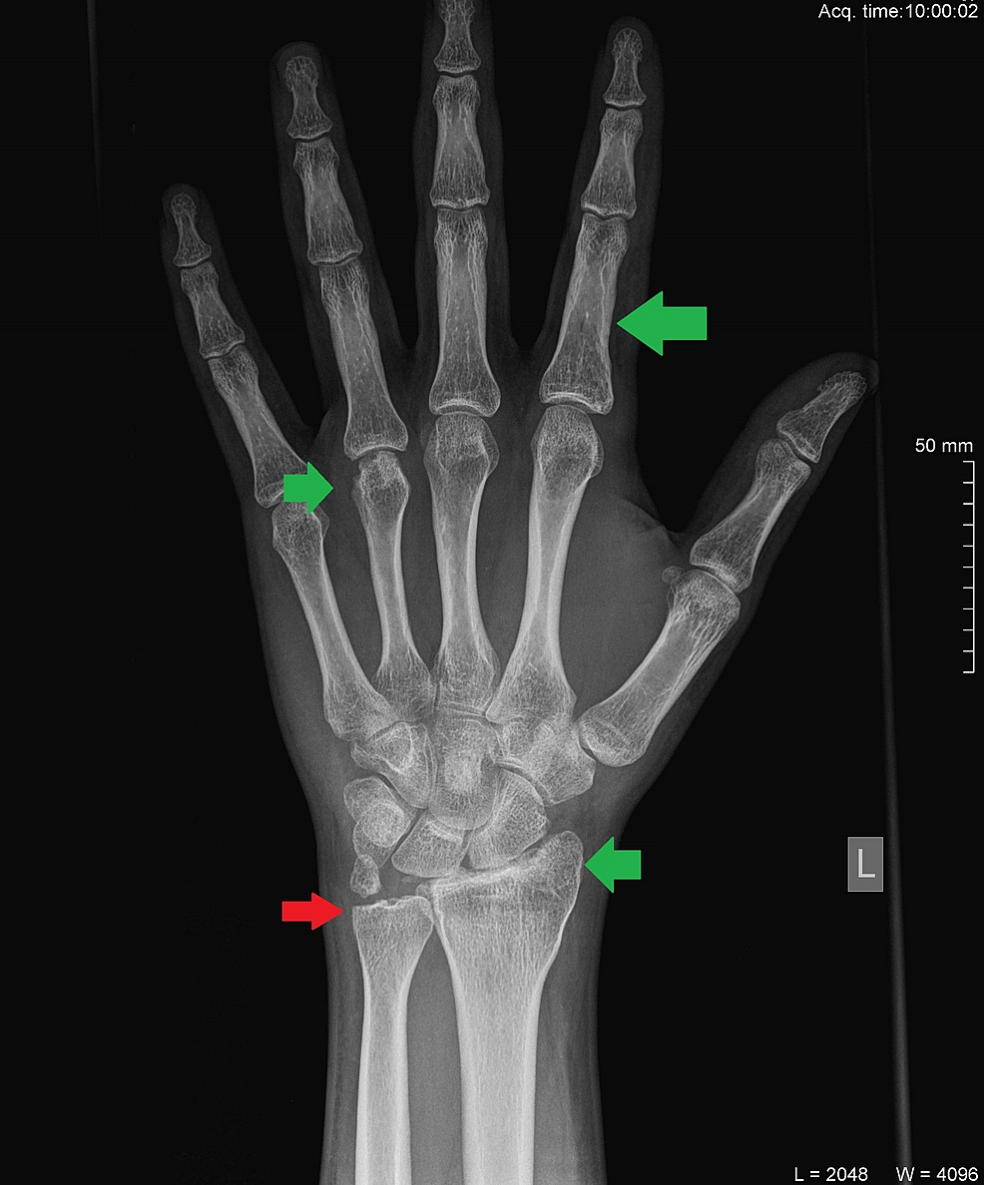
Anteroposterior radiogram of the injured wrist and hand shows the healed fractures (green arrows) and the nonhealed ulna styloid fracture (red arrow).

Conservative treatment was undertaken for the phalangeal fracture. A volar splint was placed; after postoperative day three, the patient was allowed to remove the splint and perform active flexion and passive extension exercises. At four weeks postoperatively, the splint was removed and the patient resumed progressive range of motion (ROM) and strengthening exercises. The wires were removed between the fifth and sixth week postoperatively. All fractures healed during the same period except for the ulna styloid fracture, without any functional restriction or any patient complaints (Figure 5).

**Figure 6:**
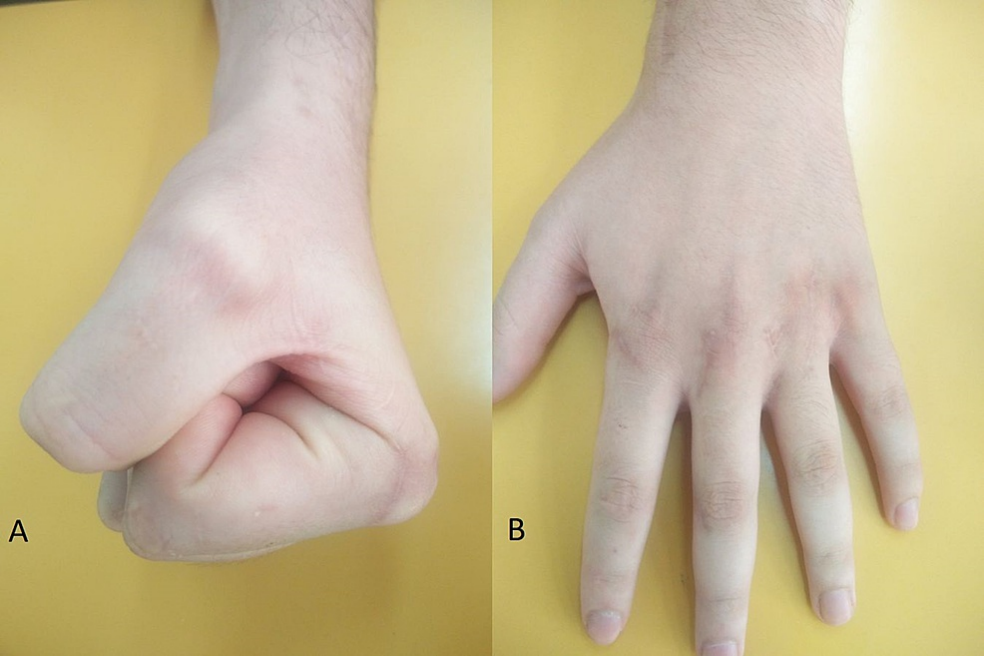
Postoperative photos at the three-month follow-up demonstrate sufficient grip strength (A) and full range of motion (A, B) of the hand.

Subsequently, the patient underwent intensive physiotherapy, and vitamin C supplementation was also prescribed. The patient regained his grip strength and full ROM gradually and returned to his daily routine three months after the initial trauma (Figures 6A, 6B).

**Figure 7:**
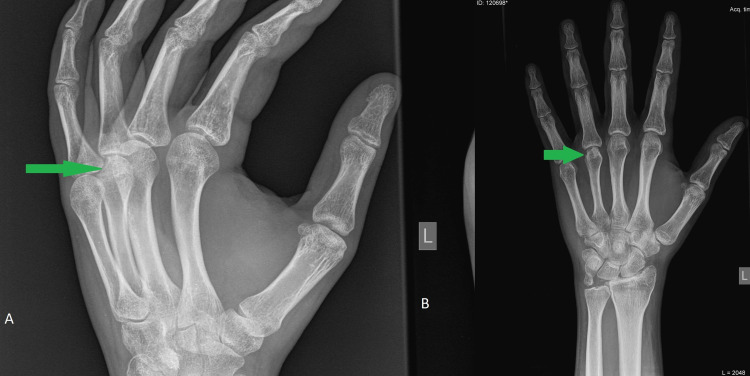
Profile (A) and anteroposterior (B) radiograms at nine months after the surgery reveal the partial avascular necrosis of the metacarpal head (green arrows).

Follow-up at nine months revealed partial avascular necrosis of the metacarpal head at the ulnar and superior side; this was, however, only a radiographic finding as the patient reported no complaints about pain or tenderness and there were no limitations in his hand functionality (Figure 7).

His grip strength was excellent and his ROM was normal. At the final 12-month follow-up, the patient reported excellent grip strength and normal ROM. In addition, the radiographs revealed unchanged avascular necrosis of the metacarpal head; hence, the patient was released from our care.

## Discussion

The combination of horizontal (transverse) metacarpal head fracture with rotation of the distal fragment followed by avascular necrosis is very rare. To our knowledge, very few cases have been reported in the literature. We present this case not only for the complexity of its management but also for the timing of the complications that may arise. It is important to maintain a high index of suspicion and monitor for complications such as avascular necrosis long after the initial injury.

Although intraarticular metacarpal head fractures are rare, their management is important as they can lead to functional impairment in the hand [6,7]. The MCP joint is a multiaxial joint permitting an arc of motion from 10 to 15 degrees of hyperextension to 110 degrees of flexion. The metacarpal head has an articular surface that is larger and longer volarly than dorsally [7]. Intraarticular MCP head fractures can be very challenging to recognize on routine radiographs. In addition to standard anteroposterior, lateral, and oblique radiographs, when such fractures are suspected, the Brewerton view is obtained (the MCP joint is flexed 60 to 70 degrees and the X-ray beam is angled 15 degrees from an ulnar to radial direction) [7,8]. Intraarticular metacarpal head fractures can be classified into 10 groups based on their anatomy: epiphyseal injuries involving the metacarpal head, osteochondral fractures, collateral ligament avulsion fractures, vertical (coronal) fractures, oblique fractures, horizontal (transverse) fractures, boxer-type metacarpal neck fractures with intraarticular split, comminuted fractures, fractures involving loss of substance, and occult fractures leading to avascular necrosis of the metacarpal head [3].

Horizontal (transverse) metacarpal head fractures are very rare and only a few cases have been reported in the literature to date. McElfresh and Dobyns reviewed 103 intraarticular metacarpal head fractures, of which only four were reported as horizontal (transverse) fractures [3]. Hastings and Carroll reported 134 closed articular fractures of the MCP and the proximal interphalangeal (PIP) joint and identified four additional horizontal (transverse) fractures [4]. Blohm and Hansen also reported a similar case [5]. Most agree that displaced MCP fractures should be treated with internal fixation using minifragment screws, Kirschner wires, pins, or minicondylar plates. The volar approach is more commonly used as it provides better visualization of the metacarpal head and the articular surface [1,6-9].

Complications include joint stiffness secondary to scarring of the ligaments, arthritis, chronic pain, and avascular necrosis [2,3,7]. Open reduction and internal fixation (ORIF) is technically difficult in some patients, and even when successful in some patients, the articular surface is under significant pressure that may lead to joint stiffness, osteoarthritis, and chronic pain. Cheah et al. reported that of 13 cases who underwent ORIF, four suffered from joint stiffness that needed arthrolysis or hardware removal [10].

Avascular necrosis of the metacarpal head is an uncommon complication of MCP fractures that is observed mostly in young adults. McElfresh and Dobyns reported four horizontal (transverse) fractures, of which three developed avascular necrosis [3]. Two of these cases had displaced and rotated fracture fragments, as in our case. The third case had a displaced fracture fragment but was not rotated. Therefore, we can conclude that horizontal (transverse) fractures result in a high degree of avascular necrosis, particularly if there is a displacement of the fracture. However, no input is provided regarding further management. It is of particular interest that the avascular necrosis became evident three to twelve months after the initial injury.

Aldekhayel et al reported another four patients with avascular necrosis of the MCP head [11]. All patients were males and had involvement of the dominant hand. Nonsurgical management was offered to all patients (rest, orthosis placement, and nonsteroidal anti-inflammatory drugs) for up to six months. Out of the four patients who suffered from avascular necrosis, two responded well to nonsurgical management, while two patients required surgical intervention with curettage and bone graft [11].

Aldekhayel et al. reviewed 40 cases (27 studies) of avascular necrosis during 1932 and 2016 [11]. The middle finger and the right hand were the most frequently affected. Clinical manifestation of avascular necrosis ranges from asymptomatic to localized pain and swelling over the MCP joint with limitation of active ROM. Other symptoms may include crepitus and reduced grip strength. Initial standard radiographs performed as diagnostic workup may reveal joint flattening, subchondral collapse, and incongruity in advanced disease [11]. Magnetic resonance imaging can also be used for detecting osteonecrosis in cases where standard radiographs fail to demonstrate pathology [12]. Treatment usually includes bone graft, curettage, debridement, vascularized joint transfer, flexion osteotomy, mosaicplasty, arthroplasty, and arthrodesis in symptomatic cases [11]. On the contrary, in our case, the fourth MCP was affected and the avascular necrosis was asymptomatic, which did not require further intervention. We hypothesized that the necrosis did not affect the MCP joint configuration but only the ulnar and upper aspect of the metacarpal head, an area that likely does not interfere majorly with the joint.

It is important to monitor avascular necrosis for up to 12 months following the initial injury as the former may present late enough to be missed. Hence, regular clinical and radiographical follow-ups may be performed, even up to one year after the injury, and intervention is needed if the avascular necrosis becomes symptomatic.

## Conclusions

Horizontal (transverse) fractures represent a rare entity that is often difficult to diagnose and has complex management. Such fractures are usually treated with internal fixation such as Kirschner wires, screws, or plates. It is essential to follow the patients even 12 months after the initial injury as late-onset complications, and especially avascular necrosis may occur. Clinical manifestations of avascular necrosis can range from pain and reduced ROM to being completely asymptomatic; usually, only symptomatic cases require intervention.
